# Immunoglobulin E and G4 epitopes of the major allergen of birch pollen Bet v 1 share residues critical for antibody binding

**DOI:** 10.1186/2045-7022-3-S3-O15

**Published:** 2013-07-25

**Authors:** N Groh, B Subbarayal, L Vogel, C Möbs, NW de Jong, W Pfützner, RG van Wijk, J Lidholm, L Meisel, S Randow, T Holzhauser, B Bohle, S Vieths, D Schiller

**Affiliations:** 1 Division of Allergology, Paul-Ehrlich-Institut, Langen, Germany; 2 Pathophysiology and Allergy Research and Christian Doppler Laboratory for Immunomodulation, Medical University of Vienna, Vienna, Austria; 3 Department of Dermatology and Allergology, Philipps University Marburg, Marburg, Germany; 4 Department of Allergology, Erasmus MC-University Medical Center, Rotterdam, the Netherlands; 5 Thermo Fisher Scientific, Uppsala, Sweden

## Background

Millions of patients with allergy to birch pollen develop clinically cross-reactive IgE against Bet v 1-like proteins in plant foods. Specific immunotherapy (SIT) with birch pollen extracts induces the biosynthesis of Bet v 1-specific immunoglobulin (Ig)G_4_. IgG_4_ is believed to act as a blocking antibody preventing IgE binding to Bet v 1, thus alleviating allergic symptoms. Only little information on the location and relationship of IgE and IgG_4_ binding sites of Bet v 1 is available. In this study we seek to identify epitopes of IgE and IgG_4_ antibodies on Bet v 1.

## Methods

A competitive immunoscreening of phage-displayed peptides was applied to predict Bet v 1 epitopes of allergen-specific IgE and IgG_4_ antibodies by bioinformatic means. Predicted epitope residues potentially critical for antibody binding were substituted by site-directed mutagenesis. Recombinant Bet v 1 (rBet v 1) and rBet v 1 variants were purified from *Escherichia coli*. The proteins were physicochemically characterized using circular dichroism (CD) and dynamic light scattering. To test the IgE and IgG_4_ interactions with rBet v 1 variants, western blot analyses, ELISA, and cellular mediator release assays were performed.

## Results

Several rBet v 1 variants were expressed in *E. coli*. Circular dichroism and structural modeling of the variants revealed Bet v 1-like theoretical secondary structure topology. The rBet v 1 variants showed reduced IgE and IgG_4_ binding with sera of birch pollen allergic subjects in western blot analyses and competitive ELISAs. The rBet v 1 variants showed decreased IgE-mediated mediator release in humanized rat basophil leukemia cells sensitized with sera of birch pollen allergic subjects.

## Conclusion

We identified critical residues in IgE and IgG_4_epitopes of Bet v 1. Although patient-specific variability was observed, the antibody interactions of the respective rBet v 1 variants were compromised for both IgE and IgG_4,_ respectively. We conclude that epitopes for IgE and IgG_4_ share common residues critical for antibody interaction, suggesting an overlap of IgE and IgG_4_-binding sites on the molecular surface of Bet v 1. The knowledge of clinically relevant immunoglobulin-allergen interactions on the molecular level enables new strategies in the diagnosis, prognosis, and therapy of both birch pollen allergies and birch pollen-related food allergies.

## Disclosure of interest

None declared.

